# Prevalence and factors associated with unhealthy metabolic status according to body mass index: analysis of a national nutritional survey

**DOI:** 10.1186/s13098-024-01411-y

**Published:** 2024-08-01

**Authors:** Víctor Juan Vera-Ponce, Fiorella E. Zuzunaga-Montoya, Luisa Erika Milagros Vásquez-Romero, Joan A. Loayza-Castro, Cori Raquel Iturregui Paucar, Enrique Vigil-Ventura, Carmen Inés Gutiérrez De Carrillo

**Affiliations:** 1 Instituto de Investigación de Enfermedades Tropicales, Universidad Nacional Toribio Rodríguez de Mendoza de Amazonas (UNTRM), Amazonas, Perú; 2 Facultad de Medicina (FAMED), Universidad Nacional Toribio Rodríguez de Mendoza de Amazonas (UNTRM), Amazonas, Perú; 3 Universidad Tecnológica del Perú, Lima, Perú

**Keywords:** Obesity, Metabolism, Epidemiologic factors, Peru (source: MeSH NLM)

## Abstract

**Introduction:**

Although obesity substantially influences public health owing to related comorbidities, it has been discovered that the incidence of such issues is not directly related to obesity but to the patient’s unhealthy metabolic status (MUS) independent of the body mass index (BMI).

**Objectives:**

To describe the prevalence of UMS overall and according to BMI and determine the factors associated with it.

**Methods:**

A cross-sectional analytical study was used based on the analysis of secondary databases called the Life Stage Nutritional Surveillance Survey (VIANEV). Participants were selected in two stages, finally obtaining 885 participants. UMS was defined based on the criteria of the Adult Treatment Panel III used to define metabolic status in a set of 5 parameters, if the subject presented two or more alterations it was considered UMS. Six groups were formed according to BMI: metabolically healthy, average weight (MHNW) and unhealthy (MUNW), metabolically healthy, overweight (MHOW) and unhealthy (MUOW), metabolically healthy, obese (MHO) and unhealthy (MUO).).

**Results:**

The total prevalence of UMS was 73.11%, with MUNW, MUOW, and MUO being 47.90%, 80.34%, and 96.44%, respectively. Only 5.31% did not present any metabolic alteration. The multivariable analysis found variations globally according to sex, age, marital status, geographical region, smoking habit, and altitude.

**Conclusions:**

A high prevalence of UMS was observed in Peru, indicating that BMI alone is not a sufficient indicator of metabolic status. These findings suggest that strategies should be prioritized to address the growing problem of UMS, considering the particularities of each subpopulation and using a multifaceted approach that addresses modifiable and non-modifiable risk factors.

## Introduction

Obesity, classically determined through the Body Mass Index (BMI) calculation, refers to an abnormal accumulation and storage of fatty tissue within the body resulting from an energy imbalance [1]. Obesity’s long-recognized substantial effects on public health, owing to related comorbid conditions, have prompted an examination of its far-reaching consequences on overall societal well-being [2]. However, it is now understood that these complications are not directly associated with obesity per se but with the patient’s unhealthy metabolic status (UMS), regardless of their BMI [3–5].

The prevalence of UMS varies according to its definition [6–10]. In the United States, it was found that 30.1% of normal-weight individuals were not metabolically healthy [11]. In Canada, research concluded that 20% of those with an average weight were metabolically abnormal due to body fat percentage [9]. In Peru, Benziger et al. discovered that those with metabolically unhealthy status, for average weight, overweight, and obesity, were 49%, 75.3%, and 96.1%, respectively.

Identifying factors related to having an unhealthy metabolic status, according to BMI, helps to pinpoint specific risk groups that could benefit from preventive interventions and precise treatments. This would allow for more effective allocation of public health resources and efforts toward populations with the greatest needs. Therefore, objectives were set to (1) describe the prevalence of UMS globally and according to BMI and (2) determine the associated factors.

## Methods

### Study design and context

This is a cross-sectional analytical study. The Life Stage Nutritional Surveillance Survey (VIANEV), conducted by the National Center for Food and Nutrition (CENAN) of Peru from 2017 to 2018, conducted a secondary analysis of the database [12]. The STROBE (Strengthening the Reporting of Observational Studies in Epidemiology) guidelines were followed for this study [13].

### Population, sample, and eligibility criteria

VIANEV collected data from three domains: Metropolitan Lima, the capital of Peru, and the rest of the urban and rural areas with a stratified, multistage, probabilistic, and independent sampling process. The sample selection underwent a two-phase process, first randomly choosing clusters as primary sampling units and then randomly selecting households containing adults aged 18 to 59 years as secondary sampling units. The intricately detailed technical report accompanying the survey includes a thorough examination of the methodology employed. Only respondents with variables that made up the metabolic status were included in this study.

### Definition of variables

UMS was defined based on the criteria established by the Adult Treatment Panel III (ATP III) [14]. To determine if a person meets this diagnosis, they must present at least two of these conditions: abdominal obesity, defined as a waist circumference (WC) ≥ 102 cm in men or ≥ 88 cm in women; hypertriglyceridemia (elevated triglyceride levels ≥ 150 mg/dl); hyperglycemia (high fasting glucose levels ≥ 100 mg/dl or if being treated to reduce glucose); elevated blood pressure (systolic blood pressure ≥ 130 mmHg or diastolic blood pressure ≥ 85 mmHg or if being treated to reduce blood pressure); low levels of HDL (HDL-cholesterol < 50 mg/dl in women or < 40 mg/dl in men).

The stratification variable was BMI, calculated as weight (kg) divided by the square of height (meters). It was divided into three groups according to the World Health Organization [15]: Normal weight (BMI less than 25 kg/m2), overweight (BMI between 25 and 29.9 kg/m2), and obesity (BMI 30 kg/m2 or higher). Thus, six groups were formed: metabolically healthy normal weight (MHNW), metabolically unhealthy normal weight (MUNW), metabolically healthy overweight (MHOW), metabolically unhealthy overweight (MUOW), metabolically healthy obesity (MHO), and metabolically unhealthy obesity (MUO).

The covariables considered as associated factors were sex (male, female), age group (categorized into 18 to 44 years and 45 to 59 years), natural region (Coast, highlands, and jungle), area of residence (urban, rural), socioeconomic level (poor, not poor), alcohol consumption in the last 30 days (yes, no), current smoking status (yes, no), physical activity level (low, medium, high), body mass index (normal weight, overweight, obesity), consumption of vegetables and fruits (< 5 and ≥ five servings per day), and altitude where they lived, measured in meters above sea level: 0 to 1499, 1500 and above.

### Data collection and procedure

Laboratory analysis necessitated patients abstain from eating for nine to twelve hours, with the minimum and maximum timeframes for fasting demarcated clearly. Then, serum was extracted and transported using a cold chain to establish the lipid profile. The measurement of triglyceride levels was performed using the automated end-point enzymatic-colorimetric method, and glucose levels were determined using pre-calibrated portable glucometers (HemoCue Glucose 201 RT).

Waist circumference was assessed upon a complete exhalation’s end using a measuring tape wrapped around the bare, upright torso with feet between 25 and 30 centimeters apart at the elevation matching the superior border of the iliac crest. This procedure was performed three consecutive times to obtain the average of the measurements.

Furthermore, the abbreviated form of the globally recognized International Physical Activity Questionnaire was employed to gauge degrees of physical movement, with categorization relying upon differentiation of low, moderate, and high levels of exercise. Poverty was evaluated using an absolute and objective monetary approach, an indicator of well-being. Households living without means to sufficiently provide necessities such as food, clothing, healthcare, and education or addressing some needs but not others were in poverty, but those enabling all fundamental requirements to be met comfortably avoided such a deprived state.

The consumption of fruits or vegetables was assessed by asking: How many servings of vegetables and fruits do you eat daily? This was categorized dichotomously into less than five servings versus five or more.

### Statistical analysis

R software version 4.0.5 was used for statistical analyses and executed considering the sample weights. Initially, a descriptive study was conducted, both univariate and bivariate, to compile categorical variables in absolute values and percentages and numerical variables in mean with standard deviation (SD). Subsequently, Poisson regression with robust variance was employed to obtain the adjusted prevalence ratios (PRa) for the covariables above and their respective confidence intervals.

Furthermore, four figures were produced. The first was a forest plot to present the prevalence of nutritional and metabolic states, respectively. The second was created to show the number of metabolic alterations (from one to five) according to BMI. The third is understanding the distributions of variables that comprise the unhealthy metabolic status according to BMI. Finally, the fourth presented a graphic map of Peru showing the degree of UMS prevalence by department.

## Results

A total of 885 subjects were included in the study. The prevalence of females was 55.59%. The percentage of individuals aged between 45 and 59 years was 33.11%. A total of 67.57% were located in urban areas. Regarding lifestyle, only 2.49% smoked daily, 49.15% had consumed alcohol in the past 20 days, and merely 39.66% consumed at least five servings of fruits/vegetables. Additionally, only 5.31% showed no metabolic alterations. The rest of the results are presented in Table 1.

**Table 1:** Descriptive characteristics of the study sample

Characteristic	*n* = 885
**Sex**	
Female	492 (55.59%)
Male	393 (44.41%)
**Age group**	
18–44 years	592 (66.89%)
45–59 years	293 (33.11%)
**Civil status**	
Single	318 (35.93%)
With couple	567 (64.07%)
**Educational Level**	
None/primary	199 (22.59%)
Secondary/Higher	682 (77.41%)
**Natural region**	
Coast	598 (67.57%)
Mountain Range	157 (17.74%)
Jungle	130 (14.69%)
**Area of residence**	
Rural	287 (32.43%)
Urban	598 (67.57%)
**Wealth index**	
Poor	164 (18.53%)
No poor	721 (81.47%)
**Alcohol consumption**	
No	450 (50.85%)
Yes	435 (49.15%)
**Daily smoking**	
No	863 (97.51%)
Yes	22 (2.49%)
**Physical activity**	
Low	587 (66.33%)
Moderate/High	298 (33.67%)
**Fruit and vegetable consumption** ** ≥ 5 servings per day**	
Less than five	534 (60.34%)
Five or more	351 (39.66%)
**Altitude level**	
0 to 1499	714 (80.68%)
1500 or more	171 (19.32%)
**Abdominal obesity**	
No	523 (59.10%)
Yes	362 (40.90%)
**Hyperglycemia**	
No	329 (37.18%)
Yes	556 (62.82%)
**High blood pressure**	
No	678 (86.04%)
Yes	110 (13.96%)
**Hypertriglyceridemia**	
No	529 (59.77%)
Yes	356 (40.23%)
**Low HDL**	
No	208 (23.50%)
Yes	677 (76.50%)
**Metabolically unhealthy for level**	
Cero	47 (5.31%)
One	191 (21.58%)
Two	271 (30.62%)
Three	256 (28.93%)
Four	106 (11.98%)
Five	14 (1.58%)
n (%)	

In the multivariable analysis, globally, factors associated with presenting UMS were being male (aPR: 0.75; 95% CI 0.68, 0.81), being in the age group of 45–59 years (aPR: 1.20; 95% CI 1.11, 1.29), and residing at altitudes of 1500 m above sea level or more (aPR: 0.81; 95% CI 0.70, 0.93). Regarding MUNW, being male (aPR: 0.68; 95% CI 0.53, 0.88), being in the age group of 45–59 years (aPR: 1.39; 95% CI 1.09, 1.77), and residing in the Jungle region (aPR: 1.50; 95% CI 1.09, 2.06) were factors. For MUOW, being male (aPR: 0.78; 95% CI 0.69, 0.87) and residing at altitudes of 1500 m or more (aPR: 0.78; 95% CI 0.64, 0.94) were factors. Lastly, for MUO, being male (aPR: 0.92; 95% CI 0.86, 0.98), residing in the Jungle (aPR: 1.04; 95% CI 1.01, 1.08), and the consumption of fruits/vegetables (aPR: 1.07; 95% CI 1.02, 1.11) were factors. (Table 2.)

**Table 2:** Regression analysisanalysis of factors associated with metabolically unhealthy state according to body mass index

Characteristic	Metabolically unhealthy		MUNW		MUOW		MUO	
	aPR*	95% CI	aPR*	95% CI	aPR*	95% CI	aPR*	95% CI
**Sex**								
Female	—	—	—	—	—	—	—	—
Male	**0.75**	**0.68**,** 0.81**	**0.68**	**0.53**,** 0.88**	**0.78**	**0.69**,** 0.87**	**0.92**	**0.86**,** 0.98**
**Age group**								
18–44 years	—	—	—	—	—	—	—	—
45–59 years	**1.2**	**1.11**,** 1.29**	**1.39**	**1.09**,** 1.77**	1.06	0.95, 1.17	1.01	0.96, 1.06
**Natural region**								
Coast	—	—	—	—	—	—	—	—
Mountain Range	0.95	0.82, 1.09	1.16	0.80, 1.68	0.98	0.82, 1.16	0.93	0.83, 1.05
Jungle	1.07	0.97, 1.19	**1.5**	**1.09**,** 2.06**	1	0.88, 1.14	**1.04**	**1.00**,** 1.08**
**Area of residence**								
Rural	—	—	—	—	—	—	—	—
Urban	0.98	0.89, 1.09	0.9	0.65, 1.26	0.95	0.85, 1.08	1.01	0.96, 1.06
**Wealth index**								
Poor	—	—	—	—	—	—	—	—
No poor	1.02	0.91, 1.14	1.08	0.79, 1.47	1	0.88, 1.13	1	0.93, 1.07
**Alcohol consumption**								
No	—	—	—	—	—	—	—	—
Yes	1.01	0.93, 1.10	1.23	0.94, 1.60	0.93	0.83, 1.03	1	0.95, 1.04
**Daily smoking**								
No	—	—	—	—	—	—	—	—
Yes	1.09	0.87, 1.36	0.97	0.49, 1.91	0.98	0.66, 1.44	1.03	0.98, 1.08
**Physical activity**								
Low	—	—	—	—	—	—	—	—
Moderate/High	1.02	0.95, 1.11	1.18	0.94, 1.50	0.94	0.84, 1.05	0.98	0.93, 1.03
**Fruit and/or vegetable consumption**								
Less than five	—	—	—	—	—	—	—	—
Five or more	0.98	0.91, 1.07	0.88	0.70, 1.12	1.04	0.94, 1.15	**1.07**	**1.00**,** 1.13**
**Altitude level**								
0 to 1499	—	—	—	—	—	—	—	—
1500 or more	**0.81**	**0.70**,** 0.93**	0.82	0.60, 1.12	**0.78**	**0.64**,** 0.94**	1.02	0.94, 1.11

aPR = Ajusted prevalence Ratio, CI = Confidence Interval

*Each factor has been adjusted independently for the rest of the variables

**Fig. 1:**
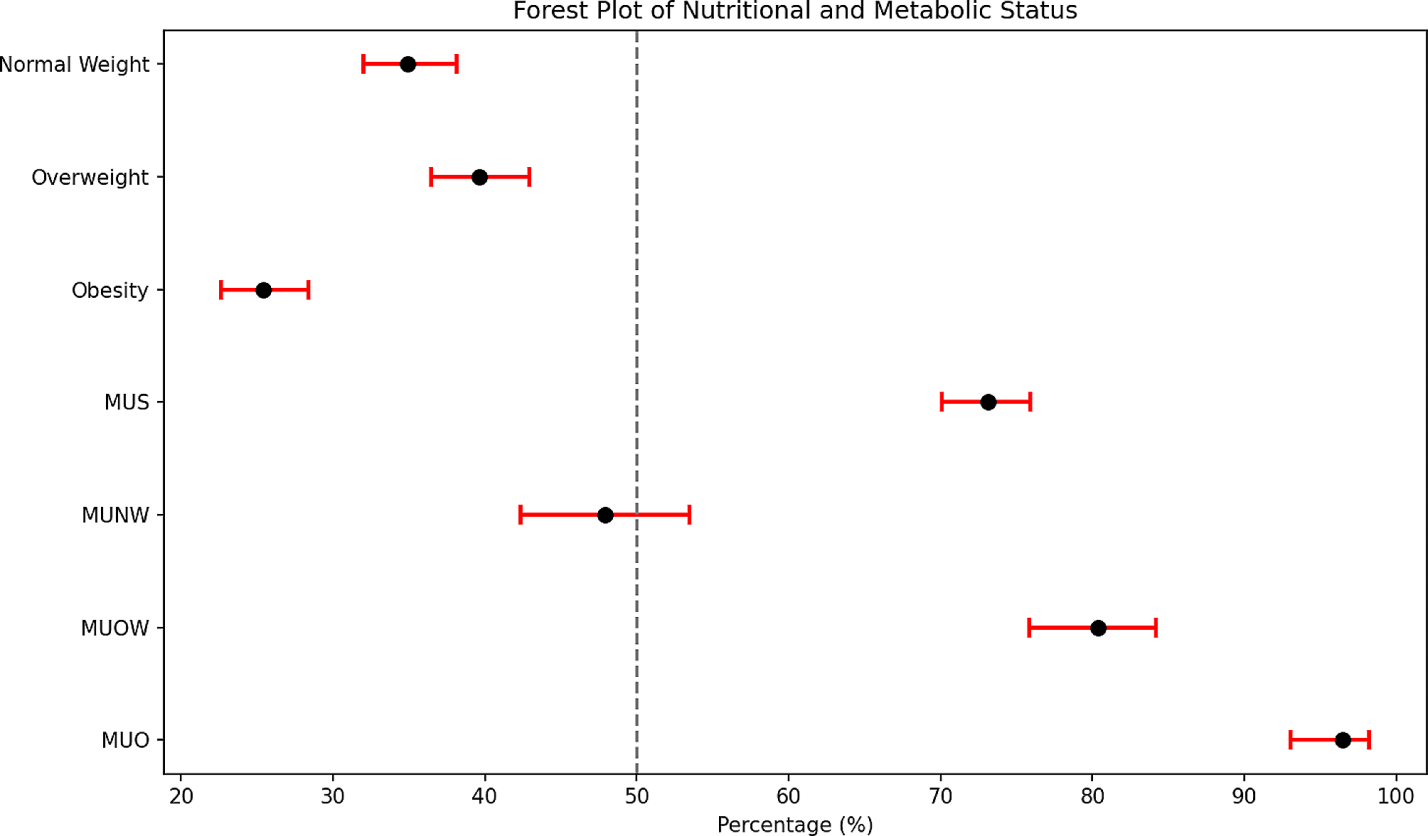
Prevalence of Nutritional and Metabolic Status

Figure 1 illustrates the total prevalence of nutritional and metabolic status. In summary, the prevalence of UMS was 73.11% (95% CI 70.06, 76.01), while the prevalence of MUNW, MUOW, and MUO were 47.90% (95% CI 42.20, 52.62), 80.34% (95% CI 75.79, 84.37), and 96.44% (95% CI 93.11, 98.45), respectively.

**Fig. 2:**
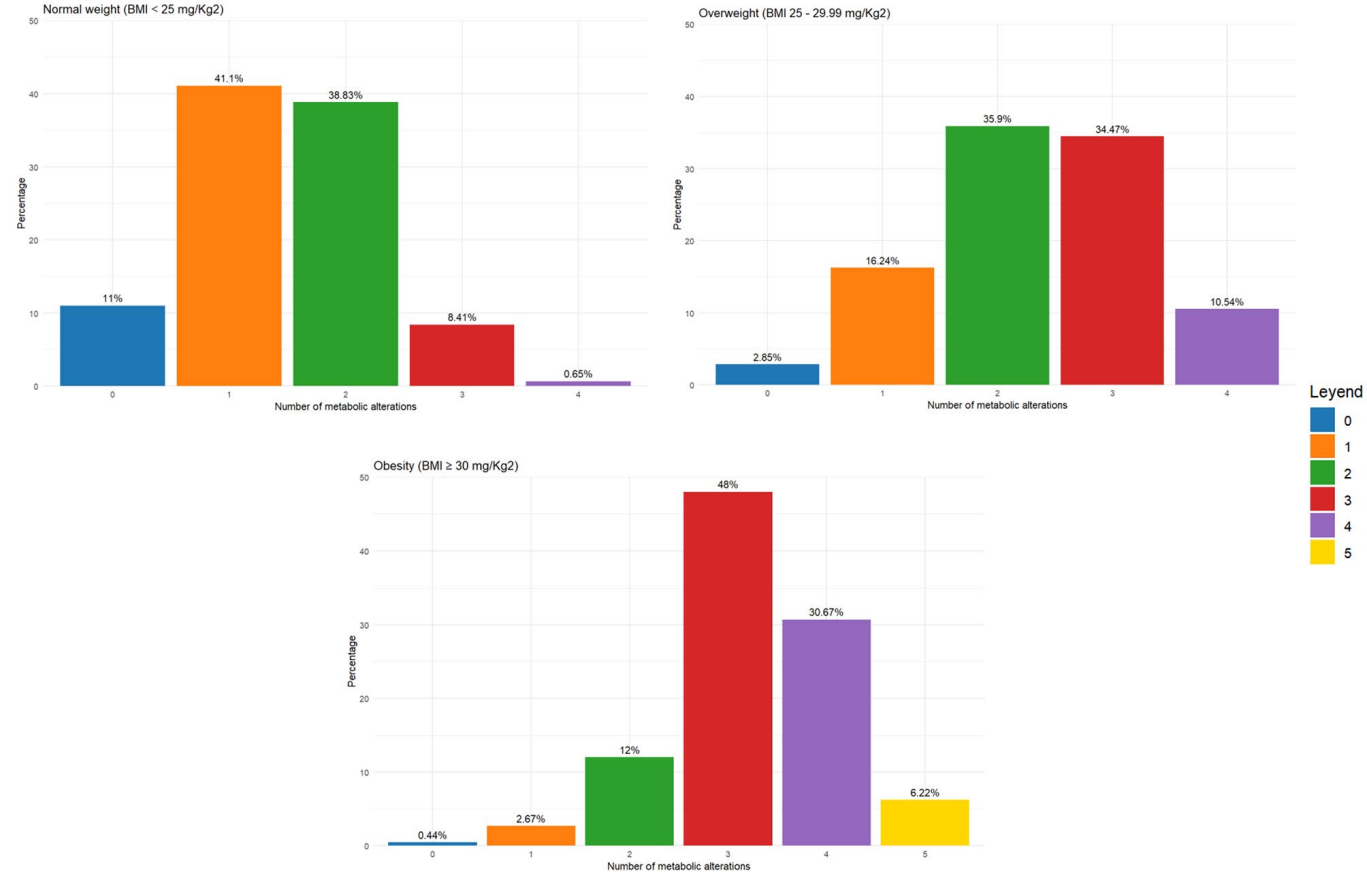
Distribution of the number of metabolic alterations according to BMI

Figure 2 shows a distribution where metabolic alterations significantly vary with BMI. Most individuals of average weight had a single metabolic alteration. In contrast, individuals with overweight and obesity exhibited a higher proportion of multiple metabolic alterations. Specifically, obesity was associated with a higher prevalence of two and three metabolic alterations, highlighting a relationship between higher BMI and more observed metabolic alterations.

**Fig. 3:**
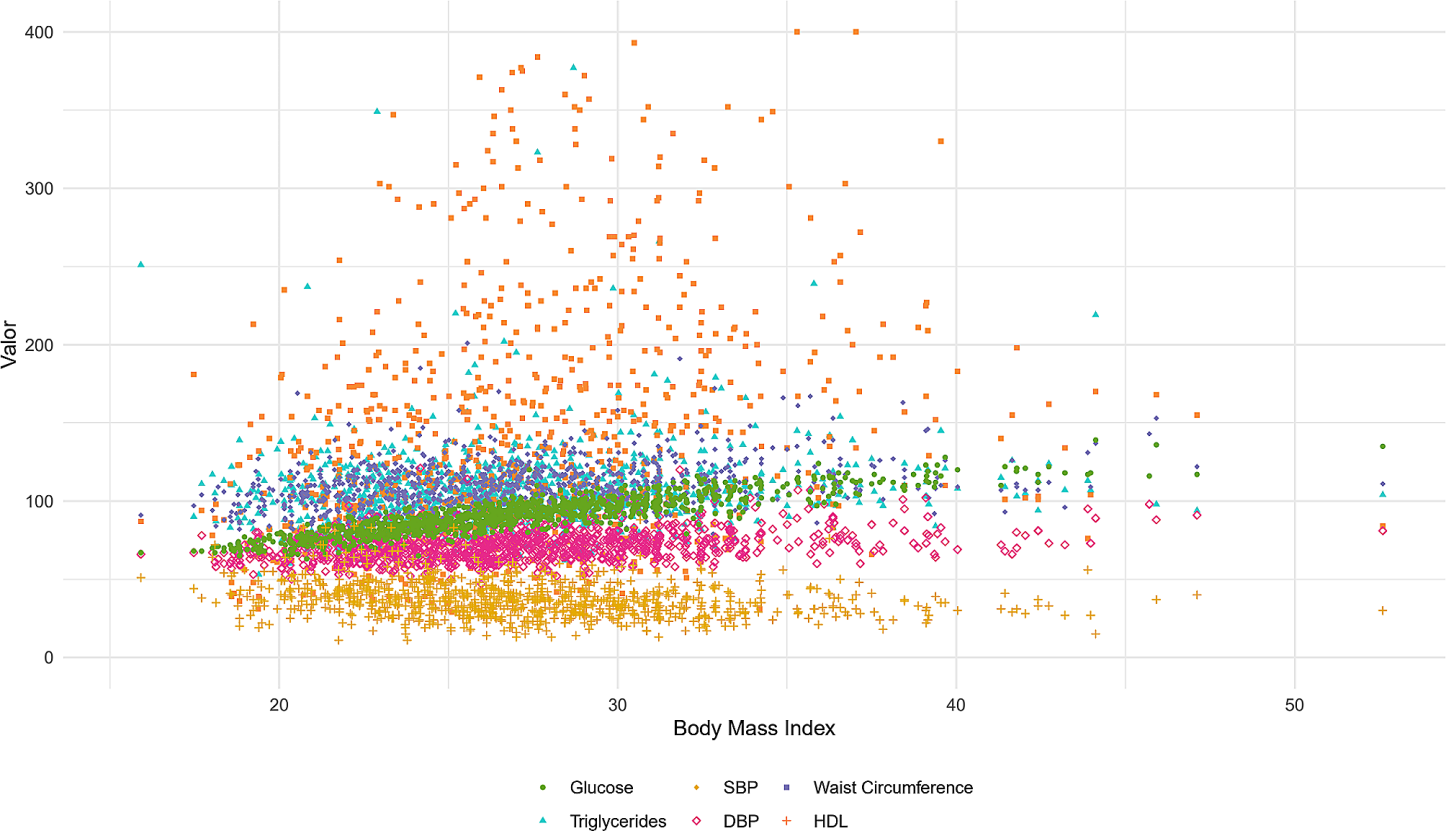
Scatter Plot of Metabolic Biomarkers as a Function of BMI

Figure 3 reveals a scatter plot suggesting the variability of each clinical measure of BMI. In particular, a broader dispersion of glucose, triglycerides, and SBP values increases as BMI increases. In contrast, HDL and WC values remain relatively constant across the BMI range, whereas the distribution of SBP is more dispersed.

**Fig. 4:**
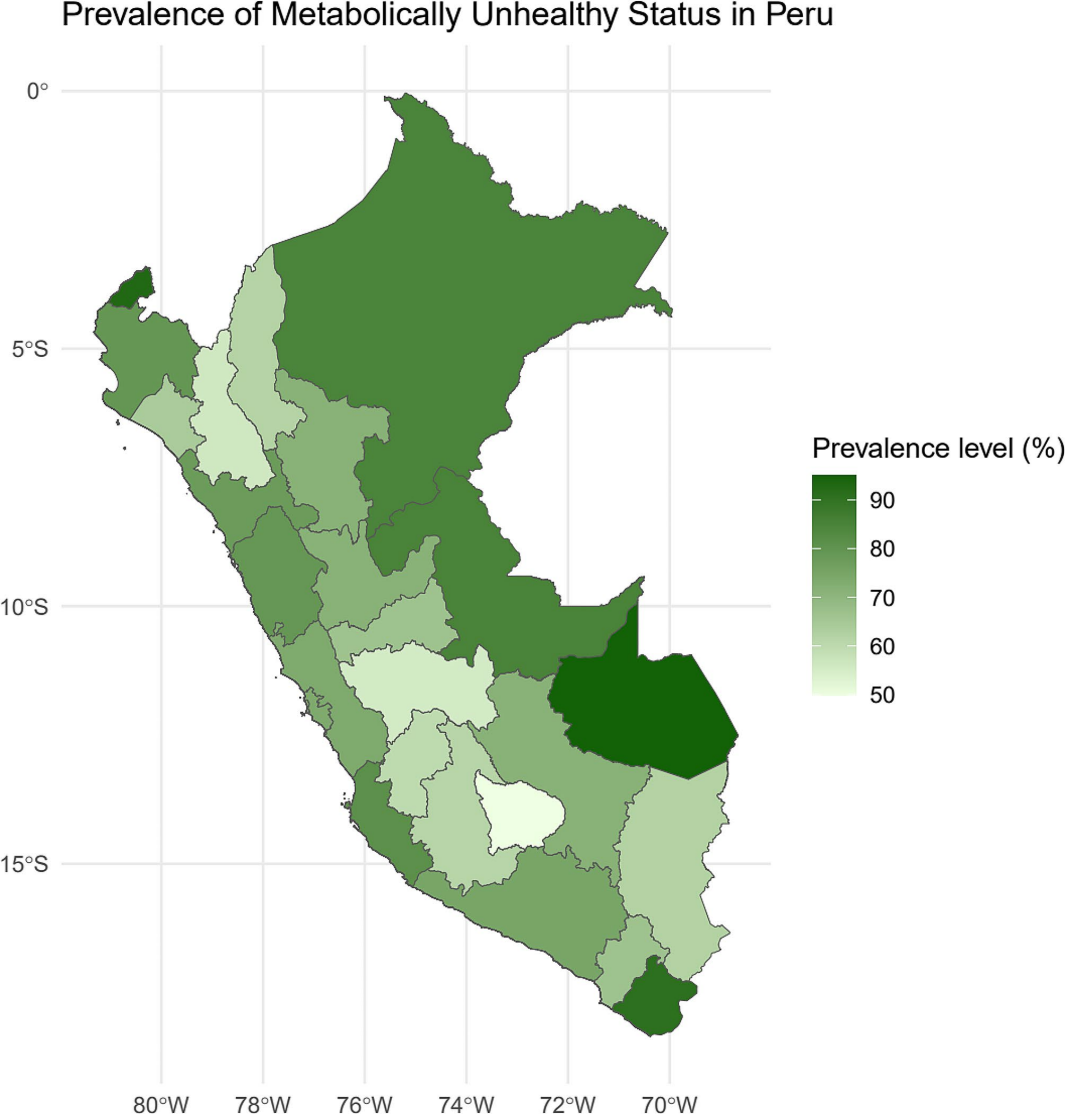
Map of Peru showing the distribution of MUS prevalence by department

Regarding the distribution of UMS prevalence by department, as graphed in Fig. 4, it was higher in the departments of Madre de Dios, Tumbes, Arequipa, and Tacna, exceeding 80%, while in Apurimac, Cajarmaca, and Junín, prevalences were around 50%.

## Discussion

### Prevalence of UMS

Our research found a total prevalence of individuals with UMS of 73.11%. This figure is alarmingly high and aligns with Benziger et al.‘s study [16], which concluded that the total population with UMS was 75.30%. Given that the latter was a semi-representative investigation and coincides with the national representativeness values found in this study, it is probable that the number of people with metabolic abnormalities is high in Peru. Globally, studies conducted in China, such as by Chen et al. [3], reported a prevalence of 42.16%, though it should be noted that they used the MetS criterion per se to define UMS and the study by Zhang Y et al. [17] with a prevalence of 52.11%. In the United States [11], the prevalence was 46%. These differences could be attributed to genetic factors, unhealthy diet and lifestyle, lower physical activity levels, socioeconomic inequalities, and cultural attitudes toward health and nutrition.

The presence of UMS by subgroups increased as BMI progressed, more prevalent in obese individuals than in those of average weight. The figures also showed that the concentrations of metabolic markers increased as BMI did. Goday et al. [9] found that 44.9% of obese patients had UMS, while only 12.9% were overweight, and 2.2% had normal weight. Wildman et al. [11] reported that 30.1% with normal weight had UMS. In our work, MUNW levels reached 47.90%, and those presenting obesity, up to 68.30%. These results align with the findings in the study by Benziger et al. [16], in which those with a metabolically unhealthy status for normal weight, overweight, and obesity were 49%, 75.3%, and 96.1%, respectively. It should be noted that some variability between criteria, in number and cut-off points, has been documented as a factor that significantly modifies the prevalence of MUNW.

On the other hand, it was found that the departments of Tumbes, Madre de Dios, and Tacna presented higher levels of UMS. This phenomenon is not isolated but part of a previously identified pattern in various country localities, where socioeconomic, environmental, and lifestyle factors seem to converge to influence the metabolic health of populations [18, 19]. Existing literature suggests that differences in the prevalence of metabolic alterations at the regional level can be attributed to multiple factors. Among them, variations in diet, access to health services, physical activity levels, and exposure to environmental pollutants stand out [20, 21]. In particular, the regions of Tumbes, Madre de Dios, and Tacna have undergone rapid socioeconomic and urban transformations, which could contribute to a shift in their inhabitants’ health and disease patterns. Additionally, the influence of geography and climate on the availability and consumption of local foods directly affects the metabolic profile of populations. On the other hand, health infrastructure and access to preventive health services vary considerably between regions, which could explain, in part, the disparities observed [22].

While there are certain similarities with other studies on the prevalence of UMS, the notably high level observed here cannot be ignored. This high percentage indicates a concerning prevalence of altered metabolic states among the Peruvian population, suggesting the possibility of a public health state that requires immediate attention. However, this observation raises two critical interpretations that must be carefully considered. On the one hand, the high prevalence of metabolic alterations could reflect a genuine health emergency, with a significant percentage of the population facing an elevated risk of developing cardiovascular diseases and other conditions associated with MetS. This underscores the need to implement intervention strategies and public health policies to prevent, detect, and manage these metabolic alterations.

On the other hand, the extensive prevalence of detected alterations also raises questions about the adequacy of the cut-off points used to define metabolic states. The possibility that current criteria are too restrictive and do not adequately reflect the metabolic risk in the Peruvian population suggests the need for a review and possible adjustment of these thresholds. This hypothesis could be further supported by noting that only 5.31% of the country has no alterations, which may not accurately reflect the country’s actual metabolic state. Therefore, adjusting the cut-off points to more precisely reflect metabolic risk could allow for earlier and more accurate identification of individuals at risk, facilitating more effective preventive interventions.

Given these findings, it becomes imperative to conduct additional research to explore the causes of the high prevalence of metabolic alterations in Peru and critically review the diagnostic criteria for MetS. This will contribute to a better understanding of metabolic health in specific contexts and guide the optimization of public health strategies to effectively combat the growing challenge of this pathology at the national level.

### Concept of UMS

The definition and conceptualization of UMS have been subjects of broad debate within the scientific community, resulting in multiple diagnostic criteria proposed by different authors and institutions. One such definition is provided by Wildman et al., where up to seven components are used, including C-reactive protein and insulin resistance (IR) [11]; however, there are at least five distinct approaches to defining UMS, ranging from a sole focus on IR to the inclusion of other relevant biological markers [23, 24].

For our study, we adhered to the classic definition of Metabolic Syndrome (MetS) according to the criteria established by the API [9], opting for a more conservative approach regarding the cut-off point for WC. Contrary to the usual recommendation of considering three or more alterations for the diagnosis of MetS, we decided that the presence of two metabolic alterations suffices to consider a positive diagnosis of the syndrome, in line with recent literature suggesting a need to reassess thresholds for earlier and more effective identification of individuals at risk. Other authors have also recommended this [10, 11, 24].

### Factors associated with UMS according to BMI

Regarding sex, there are discrepancies as to whether men or women are more prone to presenting an altered metabolic state. Some global research has found that it is the male sex that presents these alterations, especially in MUNW [25–27], while studies conducted in Peru determined that females were more prone. Various factors may cause women to be more predisposed to metabolic problems than men, regardless of BMI femenino [10, 28, 29]. One such factor is the difference in body fat distribution between women and men. Pre-menopausal women tend to develop peripheral obesity, accumulating subcutaneous fat, and men and postmenopausal women usually present central or android-type obesity, which increases the incidence of cardiovascular diseases. Additionally, the inflammatory state increases cardiovascular risk, mainly in women [30, 31].

Regarding age, it is necessary to consider the discrepancy of this result with other works. The propensity to develop MetS follows an ascending trajectory parallel to age advancement. The study by Ervin R found that the male and female population aged 40 to 59 years was approximately three times more likely than those aged 20 to 39 years to meet the diagnostic criteria for Mets [32]. This can be explained by advanced age and increased body fat, indicating that body fat levels have steadily increased over the past 30 years. Older individuals may have almost a third more fat than their younger stages [33]. Fat tissue accumulates towards the body’s center, surrounding internal organs [34].

Similar findings have been discovered in studies regarding the protective effect of altitude. Lopez-Pascual et al. [35, 36] published two studies on the prevalence of MetS, which could be associated with an individual’s habitat altitude. It was determined that those residing at high altitudes showed a reduced propensity to have MetS compared to their counterparts living in sea-level plains. The physiological mechanisms behind this phenomenon are not fully understood. Still, leptin and norepinephrine are presumed to potentially influence fluctuations in energy use and food intake experienced at high altitudes, amplifying sympathetic nervous system activity. This facilitates an increase in energy expenditure, an effect that persists even in individuals who have acclimated to such conditions. Additionally, leptin concentrations show an increase in cases of weight reduction that occur at high elevations compared to sea-level counterparts [37, 38].

### Public health importance of this study

This research focuses on how widespread UMS is in Peru, compared to figures from other countries. These findings indicate the urgent need to implement national plans to halt it, considering the apparent social and cultural gaps that may exacerbate this condition.

Moreover, metabolic status cannot be reliably inferred solely from physical appearance, as many people with average weight suffer from MetS. Consequently, public health initiatives and guidelines must emphasize the importance of considering additional metabolic indicators when comprehensively assessing the well-being of a population.

While reported differences in the frequency of UMS between sexes suggest that it may be necessary to adapt preventive and therapeutic approaches based on gender, women specifically seem to face particular metabolic difficulties related to fat deposits and inflammatory conditions, emphasizing the need to address these precise issues in public health tactics.

Although findings linking elevation and UMS are thought-provoking and suggest that high places may protect against some metabolic diseases, the exact reasons for this link remain uncertain. This discovery could pave the way for a more profound examination of how environmental and geographical conditions might shape metabolic well-being.

### Limitations of the study

Firstly, as both variables were measured simultaneously, it was impossible to establish a direct causal relationship based on our results. Longitudinal research is needed to investigate the connection between the associated factors and UMS, as more prospective studies are required. Secondly, it’s important to highlight that there was a small sample size for the MUO group, in contrast to other studies with extensive samples. Thirdly, as previously mentioned, there are various ways to evaluate UMS so that results may differ from different studies; however, the most classic criterion and only the most common variables were used to define an altered metabolic state.

## Conclusions

The study observed a high prevalence of UMS in Peru, indicating that BMI alone is not a sufficient indicator of metabolic status. Regarding the factors associated with these, variations were found according to sex, age, geographical region, and altitude of residence. These findings suggest that strategies should be prioritized to address the growing problem of UMS, considering the particularities of each subpopulation and using a multifaceted approach that addresses modifiable and non-modifiable risk factors. Additionally, an analysis of whether the classic cut-offs work adequately for the Peruvian environment should be conducted. Finally, investment in prevention, research, and education in this field will ensure the health and well-being of future generations in Peru and worldwide.

## Data Availability

The data supporting the findings of this study can be accessed by the original research paper at the following link: https://proyectos.inei.gob.pe/microdatos/.
